# Renal Vessel Extension With Cryopreserved Vascular Grafts: Overcoming Surgical Pitfalls in Living Donor Kidney Transplant

**DOI:** 10.3389/ti.2023.11060

**Published:** 2023-02-10

**Authors:** Guido Fallani, Lorenzo Maroni, Chiara Bonatti, Giorgia Comai, Marina Buzzi, Vania Cuna, Francesco Vasuri, Francesca Caputo, Enrico Prosperi, Federico Pisani, Beatrice Pisillo, Ludovica Maurino, Federica Odaldi, Valentina Rosa Bertuzzo, Francesco Tondolo, Marco Busutti, Chiara Zanfi, Massimo Del Gaudio, Gaetano La Manna, Matteo Ravaioli

**Affiliations:** ^1^ Department of Hepatobiliary Surgery and Transplantation, Policlinico S. Orsola-Malpighi, IRCCS Azienda Ospedaliero-Universitaria di Bologna, Bologna, Italy; ^2^ Department of Nephrology, Dialysis and Transplantation, Policlinico S. Orsola-Malpighi, IRCCS Azienda Ospedaliero-Universitaria di Bologna, Bologna, Italy; ^3^ Tissue Bank, Department of Immunohematology and Transfusion Medicine, Policlinico S. Orsola-Malpighi, IRCCS Azienda Ospedaliero-Universitaria di Bologna, Bologna, Italy; ^4^ Department of Pathology, Policlinico S. Orsola-Malpighi, IRCCS Azienda Ospedaliero-Universitaria di Bologna, Bologna, Italy; ^5^ Department of Experimental, Diagnostic and Specialty Medicine, University of Bologna, Bologna, Italy; ^6^ Department of Medical and Surgical Sciences, University of Bologna, Bologna, Italy

**Keywords:** living donor kidney transplant, renal vessel extension, cryopreserved vascular allografts, right kidney donation, abnormal kidney vascularization

## Abstract

In LDKT, right kidneys and kidneys with anomalous vascularization are often deferred because of concerns on complications and vascular reconstructions. To date, only few reports have examined renal vessel extension with cryopreserved vascular grafts in LDKT. The aim of this study is to investigate the effect of renal vessel extension on short-term outcomes and ischemia times in LDKT. From 2012 to 2020, recipients of LDKT with renal vessels extension were compared with standard LDKT recipients. Subset analysis of rights grafts and grafts with anomalous vascularization, with or without renal vessel extension, was performed. Recipients of LDKT with (*n* = 54) and without (*n* = 91) vascular extension experienced similar hospital stays, surgical complications and DGF rates. For grafts with multiple vessels, renal vessel extension granted a faster implantation time (44±5 vs. 72±14 min), which resulted comparable to that of standard anatomy grafts. Right kidney grafts with vascular extension had a faster implantation time compared to right kidney grafts without vascular lengthening (43±5 vs. 58±9 min), and a comparable implantation time to left kidney grafts. Renal vessel extension with cryopreserved vascular grafts allows faster implantation time in right kidney grafts or grafts with anomalous vascularization, maintaining similar surgical and functional outcomes.

## Graphical Abstract

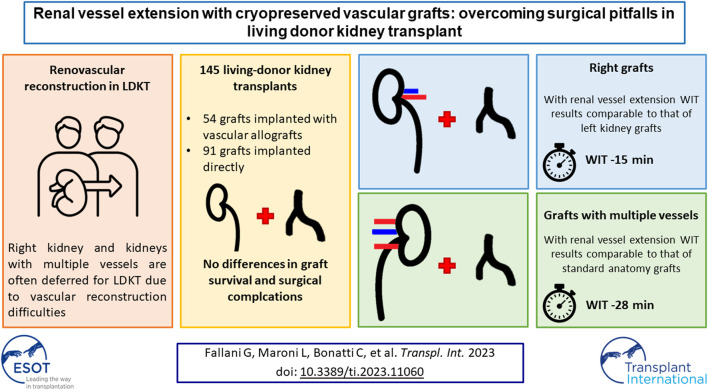

## Introduction

Kidney transplantation is the treatment of choice for end-stage renal disease (ESRD). The introduction of living donor kidney transplantation (LDKT) has allowed to extend the donor pool and to face the issues related to increasing waiting lists before transplant; nevertheless, the blanket is still short.

Although the choice of the kidney to procure should be based on “leaving the better kidney to the donor,” left donor nephrectomy is still largely predominant and often represents the default option (1). Right donor nephrectomy has been discouraged in the past, as the shorter renal vein increases the risk of renal vein thrombosis and subsequent graft loss, as well as the risk of bleeding from the inferior vena cava during procurement (2–4). A parallel surgical issue in the field of LDKT is represented by grafts with multiple arteries or veins, whose utilization has raised concerns over increased difficulty in performing more vascular anastomoses, prolonged ischemia time to at least a portion of the graft when performing more than one arterial anastomosis, poorly controlled hypertension in the post-transplant period after segmental graft ischemia or infarctions and increased rates of ureteral complications (5). Therefore, as short-term outcomes of multiple vessel renal grafts have been demonstrated worse due to higher complication and DGF rates (6), the presence of anomalous vascularization still represents a common reason to defer an organ for living donor nephrectomy.

Extension of the renal vessels during bench surgery has been advocated as a strategy to increase the straightforwardness of kidney implantation, to reconstruct multiple vessels, to facilitate positioning of the graft into the iliac fossa without kinking or twisting of the renal vessels (especially in case of obese recipients or narrow surgical field) and subsequently to reduce post-transplant complications. Different techniques have been reported in medical literature, including the use of donor gonadal vein, recipient internal iliac artery patches or latero-lateral anastomoses (7–9).

To date, few reports have addressed in detail the use of renal vessel lengthening in living donor kidney transplant. This study aims to evaluate the effects of renal vessel extension with third-party cryopreserved vascular allograft on procedural straightforwardness and short-term complications of LDKT.

## Patients and Methods

### Study Design

Consecutive recipients of living donor kidney transplantation from January 2012 to December 2020 were prospectively enrolled for the study. The population was stratified according to the use of third-party cryopreserved vascular allografts to extend the renal vessels before implantation. The clinical, demographic, and intraoperative characteristics of the two groups were compared. Subset analysis of grafts with multiple vessels, with or without vascular extension (compared to standard anatomy grafts without vascular extension as reference), and right kidney grafts with or without vascular extension (compared to left kidney grafts without vascular extension as reference) was performed to assess ischemia times and operative times.

Informed consent for study enrollment has been obtained from the subjects. The study was conducted in accordance with the principles of the 1964 Helsinki Declaration and its following revisions and was approved by the Institutional Review Board of the promoting center (Comitato Etico—Area Vasta Emilia Centro, CE-AVEC, protocol n. 312/2021/Oss/AOUBo).

### Data Collection

A database was created for the purpose of this study in a typical Excel (Microsoft Corporation—Redmond, WA, United States) spreadsheet. To ensure consistency in data entry, free-text entries were avoided as much as possible, and the admitted values for each relevant variable were restricted to a predefined cluster. Before the statistical tests were conducted the database was checked for quality, and in cases of missing, unexpected, or ambiguous data, the data were re-examined.

The data collected concerned: age, sex, comorbidities, body mass index (BMI), indications for LDKT, side and characteristics of the donated kidneys, warm and cold ischemia times, duration of surgery, postoperative complications, length of postoperative hospital stay (LOS), hospital readmissions, and postoperative mortality.

### Variable Definition, Outcome Measurements and Surgical Technique

To allow the individual risk stratification in association with concomitant diseases and physical status, the Charlson Age–Comorbidity Index (CACI) (10, 11) and the American Society of Anesthesiologists (ASA) classification (12) were used.

HLA compatibility was evaluated on loci A, B and DR as per our center policy, and graded from 0 to 6 depending on the number of compatible alleles.

Graft with multiple vessels were defined as kidneys with multiple arteries or veins that needed to be re-implanted either because they vascularized the lower pole and ureter or because their clamping during procurement demonstrated significant parenchymal ischemia; therefore, grafts with small accessory veins or arteries ligated during procurement or bench surgery were considered as standard anatomy grafts.

Vascular allografts were retrieved either from DBD or DCD by dedicated vascular surgeons and transferred to the tissue bank. After dissection and cleansing from the surrounding tissues, vessels were decontaminated in an antibiotic solution and stored in cryoprotective solution (RPMI added with 20% human albumin and 10% DMSO), then gradually cooled up to −140/−180°C. The vascular allografts were then preserved in nitrogen vapors, where they could be stored for a maximum time of 5 years, and shipped upon request to the operating room for bench reconstruction.

Left kidneys were procured with open retroperitoneal approach through a lumbar mini-incision (10 cm), while right kidneys were procured with transperitoneal approach through a right subcostal mini-laparotomy (10 cm) (13). Renal vessel extension was performed during bench surgery according to graft’s anatomy and side, to the length of the graft’s vessels and to the transplant surgeon preference. Kidney transplant was performed on the same side of the procured graft unless specific contraindications occurred (e.g., severe atheromasia of the external iliac artery, previous kidney transplant on that side or narrow surgical field due to native kidney polycystosis); all transplants in this series were performed by the same surgeon (MR). All grafts were stored in sterile ice-cold perfusion solution until implantation. Cold ischemia time (CIT) was defined as the time interval from cold *ex-vivo* flushing to the beginning of implantation. Warm ischemia time (WIT) was defined as the time interval from the beginning of implantation to reperfusion; the interval between clamping of the renal vessels in the donor and cold *ex-vivo* flushing was not accounted in the warm ischemia time as it was shorter than 2 min in all cases.

Complications were defined as any deviation from the normal postoperative course that is not inherent to the procedure and that does not imply failure to cure (14). For each patient, the postoperative complications were graded individually according to the Clavien–Dindo classification of surgical complications (14) and summarized with the Comprehensive Complication Index (CCI^®^) (15). Vascular complications were defined as any immediate or delayed complication derived from malposition, thrombosis, pseudoaneurysm, stenosis or technical failure of the vascular anastomosis that required surgical or angiographic correction.

Delayed graft function (DGF) was defined as post-transplant acute kidney injury that required dialysis in the first 7 days after transplant (16). Thirty-day acute rejection was defined as deterioration of graft function with histologically proven stigmata of rejection (according to the Banff classification of kidney allograft pathology) occurring up to POD 30 (17).

Graft loss was defined as either re-listing for transplantation or resumption of dialytic treatment.

Textbook outcome achievement was defined according to the definition proposed by Halpern et al. (18).

### Statistical Analysis

Categorical variables were presented as number and percentages, while continuous variables were presented as mean ± standard deviation or median and interquartile range (IQR) depending on their distribution. Categorical variables were compared through χ^2^ test or Fisher’s exact test depending on the numerousness of the sample, and continuous variables were compared through Student’s t test of Kruskall-Wallis one-way analysis of variance depending on their distribution. Variables with *p* < 0.10 and/or clinically relevant were put in a binary logistic regression model for multivariable analysis. Survival curves were plotted through the Kaplan-Meier estimators and compared through Log-Rank test. Differences of *p*-value <0.05 were considered significant.

All statistical analyses were performed with IBM SPSS version 26 (IBM Corporation—Armonk, NY, United States).

## Results

From January 2012 to December 2020, 145 recipients of LDKT were enrolled for the study. Overall, 43 (29.7%) right donor nephrectomies were performed. Fifty-four grafts (37.2%) underwent renal vessel extension with third-party cryopreserved vascular grafts, while 91 grafts (62.8%) were implanted without vascular extension. Among the 54 extended vessel grafts, 24 (44.4%) required the use of a venous allograft, 20 (37%) required the use of an arterial allograft and 10 (18.6%) required both venous and arterial allografts. Of the 64 vascular grafts employed, 7 (10.9%) were venous patches, 21 (32.8%) arterial patches, 27 (42.2%) were venous conduits and 9 (14.1%) arterial conduits.

### Demographics and Preoperative Characteristics

Recipients of kidneys that underwent renal vessel extension were comparable in terms of age, sex, and BMI to recipients of standard kidneys. Indications for LDKT varied between the two groups, with less cases of polycystic kidney disease and primary glomerulonephritis in the renal vessel extension group (*p* = 0.006 for all indications). Grafts with vascular extension were procured from older donors (57 ± 12 vs. 51 ± 10 years, *p* = 0.001), had a higher percentage of right kidneys (55.6% vs. 14.3%, *p* < 0.001) and more often had multiple vessels (79.6% vs. 24.2%, *p* < 0.001). Preoperative characteristics are summarized in Table 1.

**TABLE 1:** Preoperative variables.

Variables	LDKT with renal vascular extension (*n* = 54)	LDKT without renal vascular extension (*n* = 91)	*p*
Recipient age in years, mean ± SD	45 ± 14	42 ± 12	0.208
Sex			0.666
Female, n (%)	20 (37)	37 (40.7)	
Male, n (%)	34 (63)	54 (59.3)	
BMI in kg/m^2^, mean ± SD	22.9 ± 3.3	23.4 ± 3.5	0.370
Charlson Comorbidity Index, median [IQR]	0 [0–2]	0 [0–1]	0.749
Indication for KT			**0.006**
Kidney polycystic disease, n (%)	8 (14.8)	24 (26.4)	
Tubulo-interstitial nephropathy, n (%)	12 (22.2)	19 (20.9)	
Glomerulonefritis, n (%)	15 (27.8)	34 (37.4)	
Diabetic nephropathy, n (%)	1 (1.9)	3 (3.3)	
Hypertensive nephropathy, n (%)	0	3 (3.3)	
Other, n (%)	18 (33.3)	8 (8.8)	
Relationship with donor			0.197
Parent/children, n (%)	21 (38.9)	32 (35.2)	
Sibling, n (%)	10 (18.5)	28 (30.8)	
Spouse/partner, n (%)	20 (37)	30 (33)	
Other, n (%)	3 (5.6)	1 (1.1)	
Donor age in years, mean ± SD	57 ± 12	51 ± 10	**0.001**
AB0 incompatibility, n (%)	8 (14.8)	10 (11)	0.499
HLA (A/B/DR) compatibility			0.148
0, n (%)	11 (20.4)	6 (6.6)	
1, n (%)	4 (7.4)	12 (13.2)	
2, n (%)	7 (13)	12 (13.2)	
3, n (%)	22 (40.7)	33 (36.3)	
4, n (%)	6 (11.1)	12 (13.2)	
5, n (%)	2 (3.7)	6 (6.6)	
6, n (%)	2 (3.7)	10 (11)	
Pre-emptive KT, n (%)	21 (38.9)	28 (30.8)	0.318
Haemodiayisis before KT, n (%)	27 (50)	52 (57.1)	0.404
Peritoneal dialysis before KT, n (%)	9 (16.7)	21 (23.1)	0.357
Dialysis duration in years, median [IQR]	1 [0–2]	1 [0–2]	0.570
Previous KT, n (%)	5 (9.3)	6 (6.6)	0.747

Bold values highlight statistical significance.

### Intra- and Postoperative Characteristics

As grafts with vascular extension came more often from the right side, transplantation was performed more often in the right iliac fossa (53.7% vs. 24.2%, *p* < 0.001). Also, grafts with vascular allograft extension underwent longer CIT and total ischemia times (respectively 148 ± 53 vs. 107 ± 53 min, *p* < 0.001 and 192 ± 43 vs. 156 ± 52 min, *p* < 0.001); on the other side WIT was shorter in the renal vessel extension group (43 ± 5 vs. 53 ± 11 min, *p* < 0.001). Operative time was shorter in the group without vascular extension (209 ± 44 vs. 228 ± 54 min, *p* = 0.019). Intraoperative complications occurred in three cases in the group with vascular extension (5.6% vs. 0, *p* = 0.050), all unrelated to vascular anastomoses or vessel allograft utilization (one case of bleeding from a subcapsular renal hematoma, one case of bleeding from the renal hilum, one case of bleeding from the uretero-vescical anastomosis which required its remaking), as well as one case of postoperative bleeding from the arterial anastomosis (1.9% vs. 0, *p* = 0.372). Post-operative complications, PRBC transfusions, CCI^®^, delayed graft function rates, creatinine at discharge, length of hospital stay, and textbook outcome achievement rates were comparable among the two groups. Intra- and postoperative characteristics are summarized in Table 2.

**TABLE 2:** Intraoperative and postoperative variables.

Variables	LDKT with renal vascular extension (*n* = 54)	LDKT without renal vascular extension (*n* = 91)	*p*
Donated kidney side			**<0.001**
Left, n (%)	24 (44.4)	78 (85.7)	
Right, n (%)	30 (55.6)	13 (14.3)	
Multiple vessels, n (%)	40 (74.1)	6 (6.6)	**<0.001**
Multiple veins, n (%)	9 (16.7)	4 (4.4)	**0.013**
Multiple arteries, n (%)	30 (55.6)	1 (1.1)	**<0.001**
Both multiple veins and arteries, n (%)	1 (1.9)	1 (1.1)	1
Number of veins, median [range]	1 [1–3]	1 [1–2]	**0.012**
Number of arteries, median [range]	2 [1–3]	1 [1–2]	**<0.001**
Transplant side			**<0.001**
Left, n (%)	25 (46.3)	69 (75.8)	
Right, n (%)	29 (53.7)	22 (24.2)	
Induction therapy			0.455
Basiliximab and steroids, n (%)	41 (75.9)	75 (82.4)	
ATG and steroids, n (%)	11 (20.4)	15 (16.5)	
Other	2 (3.7)	1 (1.1)	
Total ischemia time, mean ± SD	192 ± 43	156 ± 52	**<0.001**
Cold ischemia time in minutes, mean ± SD	148 ± 43	107 ± 53	**<0.001**
Warm ischemia time in minutes, mean ± SD	43 ± 5	50 ± 11	**<0.001**
Operative time, mean ± SD	228 ± 54	209 ± 44	**0.019**
Intraoperative complications, n (%)	3 (5.6)	0	0.050
Postoperative complications, n (%)	20 (37)	39 (42.9)	0.490
CCI^®^, 75th percentile	20.9	8.7	0.783
Vascular complications, n (%)	1 (1.9)	0	0.372
Urinary complications, n (%)	0	0	—
ICU readmission, n (%)	1 (1.9)	5 (5.5)	0.412
DGF, n (%)	0	2 (2.2)	0.529
Creatinine at discharge in mg/dL, mean ± SD	1.29 ± 0.44	1.24 ± 0.36	0.432
30-day acute rejection, n (%)	5 (9.3)	7 (7.7)	0.762
Urinary catheter upon discharge, n (%)	0	0	—
Length of hospital stay, median [IQR]	10 [7–15]	10 [9–15]	0.163
30-day readmission, n (%)	7 (13)	20 (22)	0.178
Textbook outcome achievement, n (%)	35 (64.8)	52 (57.1)	0.362

Bold values highlight statistical significance.

### Overall and Graft Survival

**FIGURE 1:**
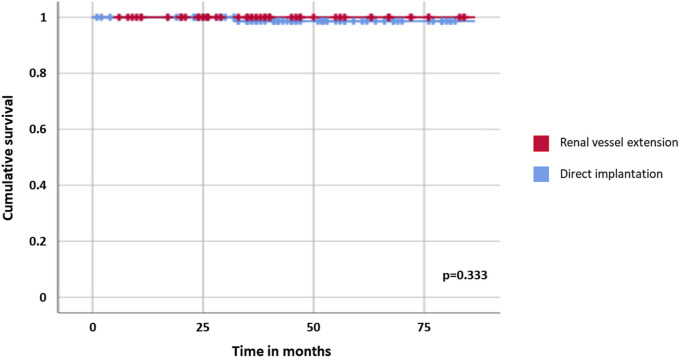
Graft survival in kidney transplants with and without renal vessel extension.

Recipients were followed for a median of 45 months after transplant [IQR: 30–71 months]; overall survival was 100% for both cohorts. Two late graft losses were observed, one for each cohort and both related to biopsy-proven primary disease recurrence on the transplanted kidney (focal segmental glomerulosclerosis in one case and IgA nephropathy in the other); graft survival curves resulted comparable between recipients of graft with and without renal vessel extension (*p* = 0.333, Table 3; Figure 1).

**TABLE 3:** Graft survival analysis between grafts with and without renal vessel extension.

Population	1-year survival rate (%)	2-year survival rate (%)	5-year survival rate (%)	*p*
Grafts with renal vessel extension	100	100	100	0.333
Grafts without renal vessel extension	100	100	98.6	

### Subset Analysis of Grafts With Multiple Vessels

Operative time, total ischemia time, CIT and WIT were compared among multiple vessel grafts with and without vascular extension (MVG-RVE and MVG), and with multiple vessel grafts with vascular extension and standard anatomy grafts without vascular extension (SAG).

Multiple vessel grafts with renal vascular extension had similar operative times compared to multiple vessel grafts without renal vascular extension (239 ± 53 vs. 225 ± 30 min, *p* = 0.542), but longer compared to standard anatomy grafts (239 ± 53 vs. 207 ± 45 min, *p* = 0.001). WIT was shorter in MVG-RVE compared to MVG and SAG (44 ± 5 vs. 77 ± 14 min, *p* < 0.001 and 44 ± 5 vs. 48 ± 8 min, *p* = 0.002, respectively), although the mean difference between MVG-RVE and SAG was only 4 minutes. CIT was longer in MVG-RVE compared both to MVG and SAG (145 ± 39 vs. 104 ± 77 min, *p* = 0.041 and 145 ± 39 vs. 107 ± 52 min, *p* < 0.001, respectively. Total ischemia time was comparable between MVG-RVE and MVG (189 ± 41 vs. 176 ± 69, *p* = 0.506), but longer in MVG-RVE compared to SAG (189 ± 41 vs. 155 ± 51 min, *p* < 0.001). These results are summarized in Table 4.

**TABLE 4:** Comparation of multiple vessel grafts with or without vascular extension and standard anatomy grafts.

Variables	Multiple vessel grafts with renal vascular extension (MVG-RVE, *n* = 40)	Multiple vessel grafts without renal vascular extension (MVG, *n* = 6)	Standard anatomy grafts (SAG, *n* = 85)	*p*	
				MVG-RVE vs. MVG	MVG-RVE vs. SAG
WIT in minutes, mean ± SD	44 ± 5	72 ± 14	48 ± 8	**<0.001**	**0.002**
CIT in minutes, mean ± SD	145 ± 39	104 ± 77	107 ± 52	**0.041**	**<0.001**
Total ischemia time in minutes, mean ± SD	189 ± 41	176 ± 69	155 ± 51	0.506	**<0.001**
Operative time in minutes, mean ± SD	239 ± 53	225 ± 30	207 ± 45	0.542	**0.001**

Bold values highlight statistical significance.

### Subset Analysis of Right Kidney Grafts

Operative time and ischemia times were also compared among right kidney grafts with and without renal vascular extension (RKG-MVE and RKG, respectively), and with left kidney grafts without vascular extension (LKG).

Operative times were comparable between right kidney grafts with renal vascular extension and right kidney grafts without renal vascular extension and group 5, and between right kidney grafts with renal vascular extension and left kidney grafts without renal vascular extension (223 ± 63 vs. 203 ± 53 min, *p* = 0.327 and 223 ± 63 vs. 209 ± 43, *p* = 0.204, respectively). WIT was shorter in RKG-RVE compared to RKG and LKG (43 ± 5 vs. 58 ± 9 min, *p* < 0.001 and 43 ± 5 vs. 48 ± 10 min, *p* = 0.014, respectively); nevertheless, the mean difference between RKG-RVE and LKG was 5 minutes. CIT was longer in RKG-RVE compared both to RKG and LKG (154 ± 50 vs. 103 ± 71 min, *p* = 0.010 and 154 ± 50 vs. 107 ± 50 min, *p* < 0.001, respectively. Total ischemia time was comparable between RKG-RVE and RKG (198 ± 50 vs. 161 ± 72, *p* = 0.064), but longer in RKG-MVE compared to LKG (198 ± 50 vs. 156 ± 48 min, *p* < 0.001). These results are summarized in Table 5.

**TABLE 5:** Comparation of right kidney grafts with or without vascular extension and left kidney grafts.

Variables	Right kidney grafts with renal vascular extension (RKG-MVE, *n* = 30)	Right kidney grafts without renal vascular extension (RKG, *n* = 13)	Left kidney grafts without renal vascular extension (LKG, *n* = 78)	*p*	
				RKG-MVE vs- RKG	RKG-MVE vs. LKG
WIT in minutes, mean ± SD	43 ± 5	58 ± 9	48 ± 10	**<0.001**	**0.014**
CIT in minutes, mean ± SD	154 ± 50	103 ± 71	107 ± 50	**0.010**	**<0.001**
Total ischemia time in minutes, mean ± SD	198 ± 50	161 ± 72	156 ± 48	0.064	**<0.001**
Operative time in minutes, mean ± SD	223 ± 63	203 ± 53	209 ± 43	0.327	0.204

Bold values highlight statistical significance.

### Multivariable Analysis of Factors Associated With Prolonged Warm Ischemia Time

As shown in Table 6, among factors possibly related to WIT (dichotomized at 45 min) only renal vessel extension resulted associated to a WIT inferior to 45 min at univariable analysis (45.7% vs. 29.3%, *p* = 0.041). Upon multivariable analysis, renal vessel extension resulted protective from prolonged WIT (OR 0.29, *p* = 0.004) while right kidney grafts resulted predictive of prolonged WIT (OR = 2.86, *p* = 0.022).

**TABLE 6:** Multivariable analysis of factors associated with prolonged implantation time.

Variables	Univariable analysis			Multivariable analysis	
	WIT≤45’ (*n* = 70)	WIT>45’ (*n* = 75)	*p*	OR [95% C.I.]	*p*
Recipient BMI in kg/m^2^, mean ± SD	23.6 ± 3.5	22.8 ± 3.4	0.172	0.91 [0.82–1.01]	0.069
Right kidney graft, n (%)	18 (25.7)	25 (33.3)	0.315	2.86 [1.17–7.00]	0.022
Multiple arteries, n (%)	17 (24.3)	14 (18.7)	0.410	2.60 [0.72–9.31]	0.143
Renal vessel extension, n (%)	32 (45.7)	22 (29.3)	**0.041**	**0.29 [0.13–0.67]**	**0.004**

## Discussion

The gap between potentially transplantable kidneys and patients with ESRD who would benefit from transplant is one of the key issues of modern time kidney transplantation and has serious consequences on the morbidity and mortality of transplant candidates (19). The progressively increasing imbalance between donors and recipients has urged to expand the donor pool, both enrolling marginal living donors and adopting techniques to recondition marginal cadaveric grafts (20, 21). LDKT is crucial for the future sustainability of kidney transplantation programs, as it both allows to relieve transplant waiting lists and grants superior outcomes for the recipients, especially in terms of reduced DGF rates (22, 23). Although the rate of living donation is constantly increasing, its proportion remains small compared to the size of waiting lists (19). Therefore, not only living donation should be promoted and given awareness, but the process of kidney procuring from living donors should be further optimized, maintaining donor safety as the key priority.

To achieve this aim, it is essential to address surgical pitfalls in living donor procurement, such as the shortness of renal vein in right donor nephrectomy and the increased WIT in grafts with anomalous vascularization. Although reports on renal vein thrombosis and arterial kinking after right LDKT are limited and often come from older studies (2–5), right donor nephrectomy still has a prevalence around 20% in most series of living donor nephrectomies and is often influenced by transplant centers expertise (13, 24, 25) Also grafts with multiple vessels still raise concern, as the necessity of multiple arterial anastomoses implies a longer WIT, whose association with delayed graft function is well established both in deceased and living donor KT (26–32). In the field of LDKT, renal vessel lengthening may represent a technical solution both for right kidney grafts with short veins and for grafts with anomalous vascularization, although most of the reports in literature are anecdotal and only few focus on technical and surgical outcomes of the procedure (7, 33, 34). Nevertheless, over the years, many different techniques have been proposed for renovascular reconstruction in LDKT (7, 8, 35). For what concerns the right renal vein, additional length might be obtained through deeper dissection of hilar structures during bench surgery, or through iliac vein transposition and eventual internal iliac vein ligation. On the other hand, multiple renal vessels are commonly reconstructed during bench surgery through end-to-side or pantaloon anastomoses. The choice to perform one type of reconstruction or another is usually dependent on the spatial configuration of the vessels, but surgeon’s experience and preferences have a relevant impact in the decision making. The comparation of different techniques to achieve more length or less anastomoses on the surgical field is far from the intent of this study; however, it is undoubtable that both renal vessel extension with vascular grafts (either autologous or heterologous) and deeper surgical dissection (either on the recipient or on the graft during bench surgery) carry a risk of complications (e.g., bleeding, thrombosis of the anastomoses, unnoticed lesions on the graft pelvis).

The results of this study show that renal vessel extension with cryopreserved vascular grafts allows to perform LDKT with right kidney or multiple vessel grafts granting comparable warm ischemia time to left or standard anatomy grafts, without increasing vascular complications or DGF rates in the recipients. The first obvious result from the analysis of data is the shorter warm ischemia time in the group of grafts with vessel lengthening (43 ± 5 vs. 50 ± 11 min, *p* < 0.001), although a mean difference of 7 minutes may not imply a true clinical difference. As expected, grafts with renal vessel extension have undergone a longer cold ischemia time, possibly related to a longer bench surgery, and a longer total ischemia time. For what concerns operative time, transplants of kidney grafts with vascular extension had longer operative times: this result may be consequent to a more accurate positioning of the graft after completion of the anastomoses, to avoid kinking of the longer reconstructed renal vessels.

**FIGURE 2:**
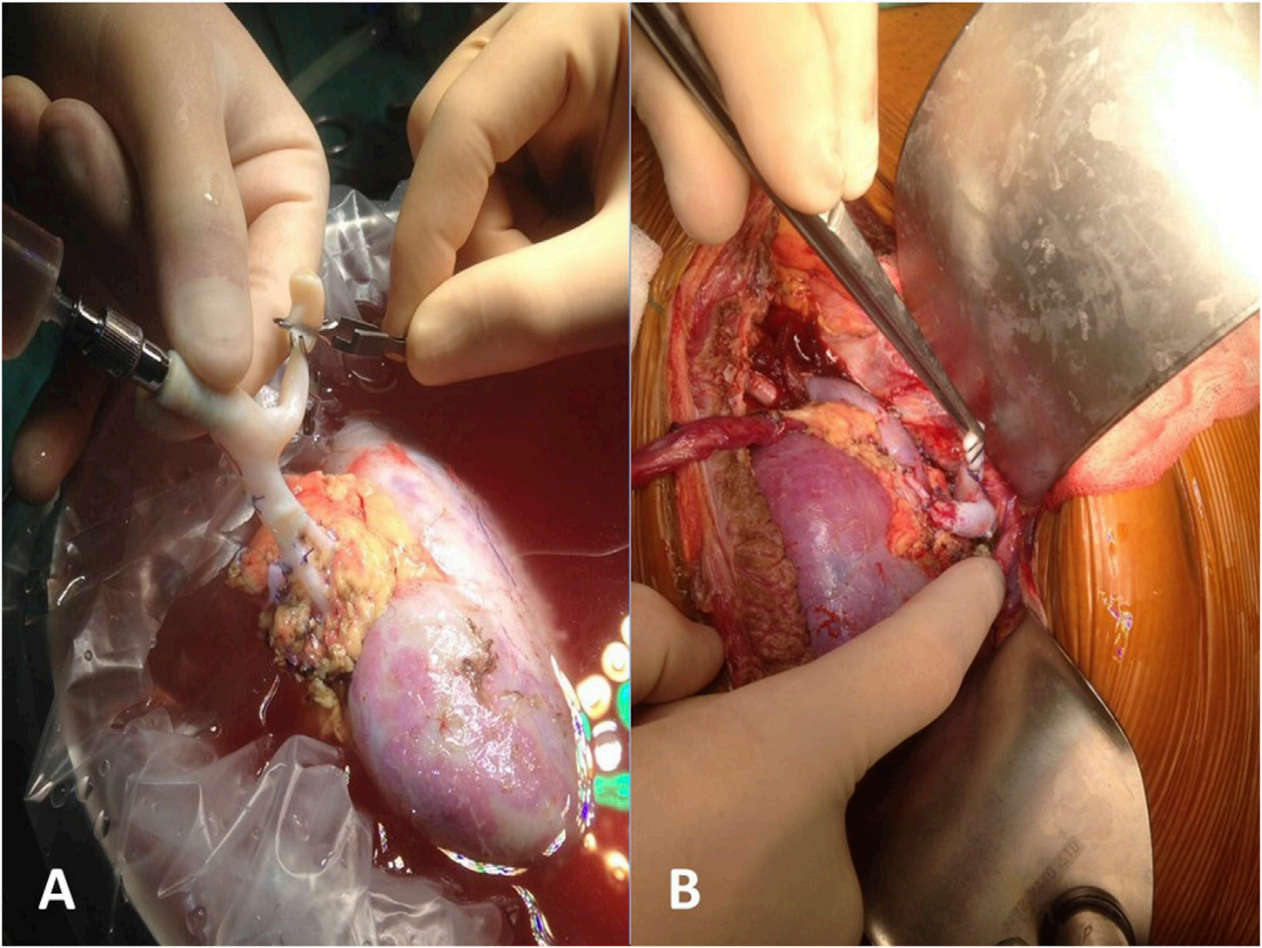
Reconstruction of multiple arteries with a single cryopreserved vascular graft: bench surgery **(A)** and reimplantation **(B)**.

Focusing on grafts with multiple vessels, the use of renal vessel extension techniques was demonstrated to be associated to a reduced WIT compared both with multiple vessels grafts without vascular lengthening (44 ± 5 vs. 72 ± 14 min) and to standard anatomy grafts without vascular lengthening (44 ± 5 vs. 48 ± 8 min), although the mean difference of 4 min with the latter may not imply a clinical significance. Nevertheless, the use of cryopreserved vascular grafts granted the possibility to perform single vascular anastomoses, thus determining warm ischemia times comparable to those of standard anatomy grafts (Figure 2). This result has paramount importance given the known detrimental effects of total warm ischemia time and implantation time on graft function (26–32), and the association between multiple arteries and longer WIT [6, 32]. As expectable, renal vessel extension implied a longer CIT, both compared to multiple vessel graft without lengthening and to standard anatomy grafts; nevertheless, total ischemia time in multiple vessel grafts with and without vascular lengthening was comparable, as the time spent on bench surgery was regained through a faster implantation time. Operative time was comparable for grafts with multiple vessels regardless of vascular lengthening procedures, but shorter compared to standard anatomy grafts; this is probably due to the time spent in positioning the graft after implantation, which represents a key step of the intervention whether there are extended vessels or multiple vessels without extension.

**FIGURE 3:**
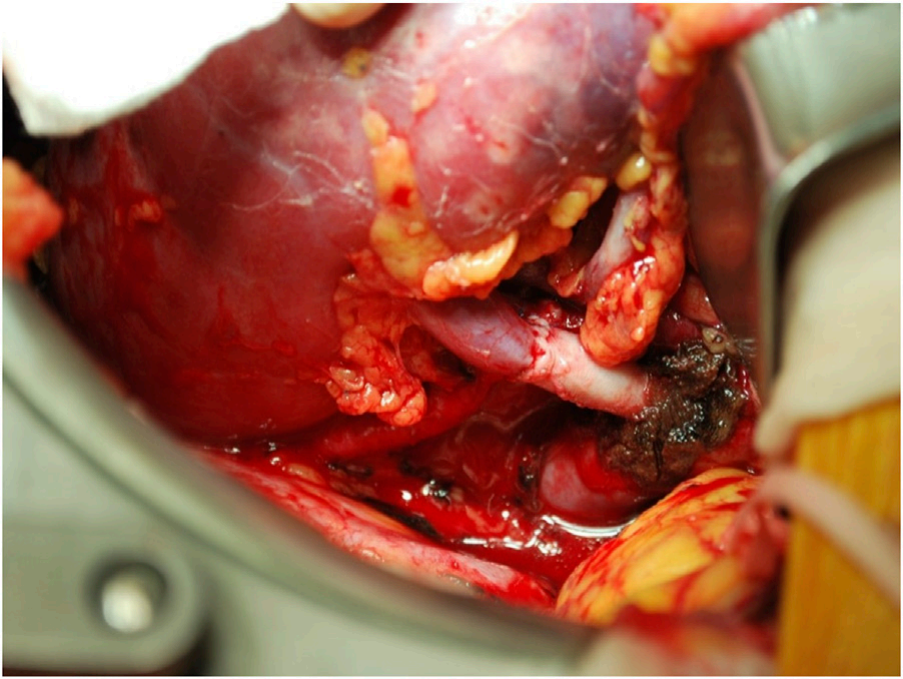
Extension of right renal vein through cryopreserved vascular graft.

For what concerns right kidney grafts, it is notable that in this case series the percentage of right donor nephrectomies (43/145, 29.7%) was above the majority of those previously reported in medical literature, which lays around 20% (5, 24, 25). The subanalysis of right kidney grafts showed that renal vessel extension was associated with shorter WIT, both compared to right grafts without vascular lengthening (43 ± 5 vs. 58 ± 9 min) and to left grafts (43 ± 5 vs. 48 ± 10 min); also in this case, the mean difference of 5 min between right kidney grafts with vascular extension and left kidney grafts may not imply an actual clinical significance. It is however evident that an extended renal vein allows for a bigger surgical field (Figure 3), and subsequently a more straightforward implantation, which by these results is comparable to that of left kidney grafts. Again, cold ischemia time was longer for right kidney grafts with vascular extension compared to standard kidney grafts and left kidney grafts; however, total ischemia time resulted comparable among right kidney grafts with and without vascular lengthening, stating that the faster implantation time in grafts with vascular extension allowed to retrieve the adjunctive time spent during bench surgery. Since the first LDKT in 1954, more than half a million donor nephrectomies have been performed, and—although selection and management of the donors have improved substantially—kidney donors still carry an increased risk of end-stage renal disease (36, 37). In the process of LDKT, donor short- and long-term outcomes must be guaranteed at all costs, and—given the risk of renal failure after donation—the choice of the kidney to donate should not be affected by technical issues. As per the results of this study, renal vessel extension appears to be a useful tool to address surgical pitfalls in LDKT with right kidney grafts or grafts with anomalous vascularization, allowing to facilitate the vascular anastomoses and to reduce implantation time, making it comparable to left/standard anatomy grafts without compromising graft function and short-term outcomes. Notably, in this study only the use of right kidney grafts resulted predictive of a longer implantation time, while the use of renal vessel extension techniques was proved to be protective from a longer implantation time. Also, recipient BMI and anomalous vascularization, which have been reported as predictors of a longer WIT in a retrospective study by Hellegering et al. (32), did not result predictive of a prolonged implantation time in this case series, probably due to the small numbers and to the mitigating effect of routine use of third-party vascular allografts to lengthen the renal vessels. However, in clinical practice it is not uncommon to encounter the case of a potential donor with left kidney aberrant vascularization and a standard anatomy right kidney. In those cases, our experience suggests procuring the right kidney rather than the left, as right renal vein extension is technically simpler than reconstructing multiple vessels, and it allows similar results in terms of implantation time.

Finally, despite possible concerns on immunological sensitization due to third-party vascular allograft utilization [related to both HLA- and AB0-incompatibility)], in our series we did not observe any delayed vascular complication (i.e., arterial stenosis).

This study has some limitations. First, its prospective case–cohort design and the limited number of patients affected our ability to draw definite conclusions. Also, lack of data regarding the length of the procured renal vessels has limited the possibility to conduct a more precise analysis on variables associated with renal vessel extension procedures. Finally, although all transplants have been performed by the same surgeon (MR), the variability of techniques used for renal vessel extension may have impacted on the final results of the study.

## Conclusion

Living donation is a fundamental tool to provide sustainability to kidney transplantation programs, and also to grant better functional and long-term results to the recipients. As of today, anomalous kidney vascularization and short right renal veins still represent an issue in the field of LDKT, being often key factors in the process of choosing which kidney to donate, together with parameters of donor kidney function. Renal vessel extension through third-party vascular allografts represents a useful mean to address surgical pitfalls of LDKT with right grafts or grafts with anomalous vascularization, allowing a shorter implantation time while maintaining adequate functional and surgical short-term outcomes in the recipients.

## Data Availability

The raw data supporting the conclusion of this article will be made available by the authors, without undue reservation.

## Abbreviations

ASA, American society of anesthesiologists; BMI, body mass index; CACI, Charlson age-comorbidity index; CCI^®^, comprehensive complication index^®^; CIT, cold ischemia time; DBD, donor after brain death; DCD, donor after cardiac death; DGF, delayed graft function; DMSO, dimethyl sulfoxide; ESRD, end-stage renal disease; HLA, human leukocyte antigen; LDKT, living donor kidney transplant; LOS, length of stay; POD, postoperative day; PRBC, packed red blood cells; RPMI, Roswell Park Memorial Institute growth medium; WIT, warm ischemia time.
